# Characterization of VCAM-1-Binding Peptide-Functionalized Quantum Dots for Molecular Imaging of Inflamed Endothelium

**DOI:** 10.1371/journal.pone.0083805

**Published:** 2013-12-31

**Authors:** Yun Chen, Mátyás Molnár, Li Li, Peter Friberg, Li-Ming Gan, Hjalmar Brismar, Ying Fu

**Affiliations:** 1 Department of Molecular and Clinical Medicine/Clinical Physiology, The Sahlgrenska Academy and University Hospital, University of Gothenburg, Gothenburg, Sweden; 2 Science for Life Laboratory, Department of Applied Physics, Royal Institute of Technology, Stockholm, Sweden; 3 AstraZeneca R&D, Mölndal, Sweden; Brigham and Women's Hospital, United States of America

## Abstract

Inflammation-induced activation of endothelium constitutes one of the earliest changes during atherogenesis. New imaging techniques that allow detecting activated endothelial cells can improve the identification of persons at high cardiovascular risk in early stages. Quantum dots (QDs) have attractive optical properties such as bright fluorescence and high photostability, and have been increasingly studied and developed for bio-imaging and bio-targeting applications. We report here the development of vascular cell adhesion molecule-1 binding peptide (VCAM-1 binding peptide) functionalized QDs (VQDs) from amino QDs. It was found that the QD fluorescence signal in tumor necrosis factor 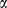 (TNF-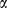) treated endothelial cells *in vitro* was significantly higher when these cells were labeled with VQDs than amino QDs. The VQD labeling of TNF-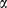-treated endothelial cells was VCAM-1 specific since pre-incubation with recombinant VCAM-1 blocked cells' uptake of VQDs. Our *ex vivo* and *in vivo* experiments showed that in the inflamed endothelium, QD fluorescence signal from VQDs was also much stronger than that of amino QDs. Moreover, we observed that the QD fluorescence peak was significantly blue-shifted after VQDs interacted with aortic endothelial cells *in vivo* and *in vitro*. A similar blue-shift was observed after VQDs were incubated with recombinant VCAM-1 in tube. We anticipate that the specific interaction between VQDs and VCAM-1 and the blue-shift of the QD fluorescence peak can be very useful for VCAM-1 detection *in vivo*.

## Introduction

Atherosclerosis develops over decades and is often silent until an acute event occurs in later life. Despite huge efforts of research on both diagnosis and therapy, atherosclerosis related cardiovascular disease (CVD) remains one of the leading causes of mortality. The most effective way of preventing and intervening CVD is to be able to diagnose first signs of CVD. In the early stages of atherosclerosis, the endothelial layer lining the lumen of the vessel undergoes a series of changes that are activated by inflammation, e.g., the increased expression of adhesion molecules at the endothelial cell surface [1], [2]. Adhesion molecules, such as vascular cell adhesion molecule 1 (VCAM-1), play an important role in the rolling and tethering of leukocytes on the vessel wall and the subsequent accumulation of macrophage-derived foam cells and the formation of atherosclerotic lesion in the vessel wall [3]. This provides a basis for a new approach to develop imaging agents that can be specific for abnormal endothelial cells, thus offering excellent opportunities for molecular imaging of early atherosclerosis. Fluorophores and magnetic particles functionalized with VCAM-1 binding peptide or anti-VCAM-1 antibody have already been used to visualize the VCAM-1 expressing endothelium [4]–[6].

Quantum dots (QDs) are semiconductor nanocrystals, normally in the diametric range of 2–20 nm, with tunable fluorescence wavelengths which depend on QDs geometric sizes. They have many attractive optical properties such as high photo- and chemical-stabilities, broad excitation spectra, narrow and symmetric fluorescence peaks, and high fluorescence efficiencies [7]–[10]. Because of these advantages, there has been a great interest in developing QDs for labeling and bioimaging [11]. QDs have been functionalized by various surface chemistry methods with specific targeting molecules such as oligonucleotides and antibodies [12], [13] for imaging cells [14], tissues [15], [16], whole animals [17], as well as for detecting diseases [18]–[21].

In our early studies where we used un-functionalized QDs to label rat endothelial progenitor cells (EPCs) [22], we found a blue-shift in the fluorescence peak of QDs located inside EPCs as compared with QDs located outside EPCs [23]. Other studies have reported the modifications of the QD fluorescence peak in different circumstances, such as ion concentrations in the surrounding media [24], DNA hybridization [25] and long-time *in vivo* persistence in animal [26].

Here we report the functionalization of QDs using a previously well-characterized VCAM-1 binding peptides [5], the binding of these functionalized QDs with VCAM-1-expressing endothelial cells, and the characterization of the fluorescence spectra of the functionalized QDs. We tested the VCAM-1 binding peptide conjugated QDs (referred hereby as VQDs) in cultured mouse endothelial cells treated with tumor necrosis factor 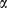 (TNF-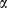), which are known to increase VCAM-1 expression in these cells [27]. We further evaluated the VQDs in a mouse model with an increased endothelial expression of VCAM-1 induced by lipopolysaccharide (LPS) [28]. It was found that VQDs can selectively target VCAM-1-expressing endothelial cells, and the QD fluorescence peak shows a significant blue-shift after VQDs interact with VCAM-1. All these can be very useful for applying VQDs to specifically detect VCAM-1 *in vivo*.

## Materials and Methods

### Functionalize QDs with VCAM-1 binding peptides

**Figure 1:**
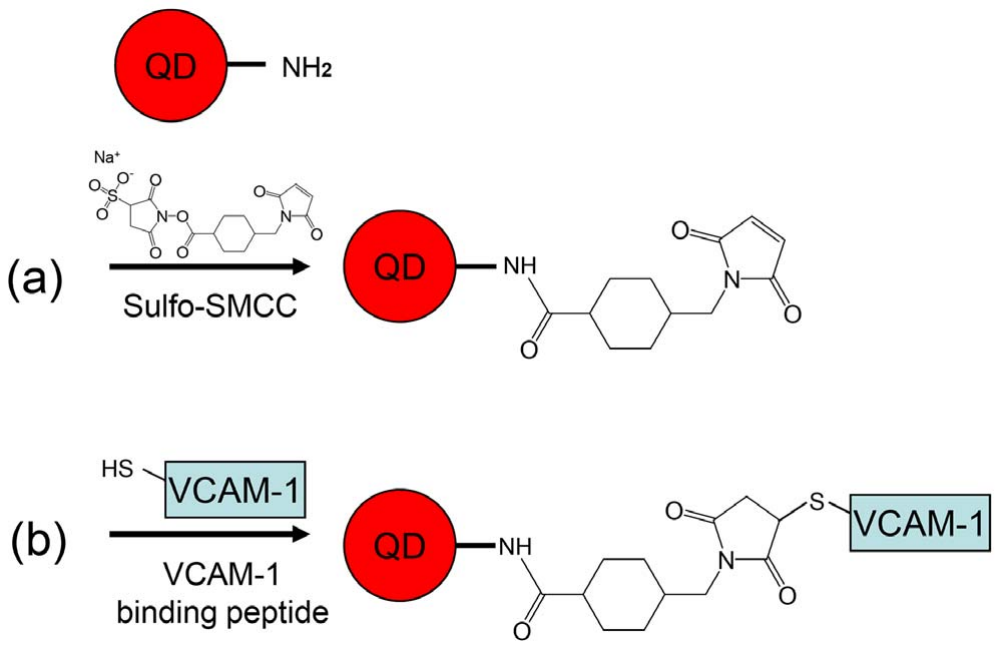
Conjugation reactions of amino QD with VCAM-1 binding peptide. (a) The first step, amino QDs are maleinated with sulfo-SMCC; (b) The second step, VCAM-1 binding peptide is attached to the QD-maleimide with thioether group.

Amino-polyethylene-glycol-coated QDs were purchased from Invitrogen, Sweden (Qdot 705 ITK™ Amino (PEG) Quantum Dots, refered hereby as amino QDs). The amino QD consists of a CdSeTe core and a ZnS shell with an actually measured fluorescence peak at about 696 nm. The amino QDs were re-suspended in 50 mM phosphate-buffered saline (PBS, Gibco, Sweden), centrifuged in Amicon Ultra-4 with 100 kDa cutoff filter (Millipore, Sweden) at 4000 g for 8 minutes, and activated with sulfosuccinimidyl-4-(N-maleimidomethyl)cyclohexane-1-carboxylate (sulfo-SMCC, Thermo Scientific, Sweden), which is a heterobifunctional crosslinker that contains N-hydroxysuccinimide (NHS) ester and maleimide groups. NHS ester reacted with primary amines at pH 7–9 to form QD-maleimides, see Fig. 1(a), thereafter QD-maleimides reacted with the sulfhydryl groups of the cysteine in VCAM-1 binding peptides at pH 6.5–7.5 to form QD-VCAM-1 binding peptides, i.e., VQDs, see Fig. 1(b) [29], [30].

Sulfo-SMCC was dissolved in 50 mM PBS to a final concentration of 5 mM. To activate QD surfaces, 100 µL sulfo-SMCC was incubated with 0.0034 nmol amino QDs at room temperature with gentle shaking. After 1 h, unattached sulfo-SMCC was removed using NAP-10 column (GE Healthcare, Sweden). The eluent was collected in eppendorf tubes: Two drops of the eluent in each tube; 5–6 deepest-colored (high QD concentration) fractions were poured together and used for the following conjugation reaction.

After removing excess sulfo-SMCC with NAP-10 column, VCAM-1 binding peptides (VHPKQHRGGSKGC, 400 nmol, Innovagen, Sweden) were added to the QD-maleimide solution and incubated overnight at 4°C with gentle shaking, resulting in the desired VQDs. Un-conjugated peptides were removed using Amicon Ultra-4 filters (100 kDa cutoff). To gain a visual evidence for the success of the conjugation reaction, the same conjugation protocol was used for conjugating QDs to 5-Carboxyfluorescein (5-FAM) labeled VCAM-1 binding peptides (Innovagen, Sweden), referred to as (5-FAM)-VQDs. The concentrations of VQDs and (5-FAM)-VQDs were determined by absorbance spectroscopy (Lamber-Beer law).

### Agarose gel electrophoresis

Agarose gel electrophoresis was performed to check the conjugation between QDs and VCAM-1 binding peptides. Following samples were added to 2 µL loading dye and 9 µL Tris-acetate-EDTA (TAE) buffer (pH 8) then loaded into 0.5% agarose gel: 1 µL amino QDs (8 µM); 4 µL QD-maleimide (2 µM); 1 µL VQDs (4 µM); 1 µL (5-FAM)-VQDs (4 µM), and 1 µL (5-FAM)-VCAM-1 binding peptides. The gel was run at a voltage of 100 V for 1.5 h.

### In vitro study with cultured endothelial cells

Mouse aortic endothelial cells (Dominion Pharmakine, S.L., Italy) were cultivated in endothelial growth medium-2 (EGM-2) containing endothelial cell basal medium-2, SingleQuots (Lonza, Denmark), 20% fetal bovine serum (FBS), 100 U/mL penicillin, 100 µg/mL streptomycin, 29.2 µg/mL glutamine, and 0.25 µg/mL amphotericin B (all from Invitrogen, Sweden), at 37°C in a humidified atmosphere with 5% CO_2_ in air. Cells (10000/well) were seeded onto a cover glass placed inside each well of a 48-well plate and incubated for 24 hours to allow cell attachment before treated with 20 ng/mL TNF-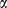 for 24 hours. After removing TNF-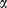, the cells were incubated with 1.3 nM amino QDs or 1.3 nM VQDs for 1, 5 or 24 hours. Cells without TNF-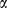 treatment served as control. To check the specificity of the VQDs for VCAM-1, VQDs were pre-incubated for 5 hours with recombinant VCAM-1 (R&D Systems, Sweden) in different ratios before adding to the cells. At the ends of incubations with amino QDs or VQDs, the cells on cover slips were washed with PBS, fixed using 4% paraformaldehyde and mounted on glass slides using Prolong Gold Antifade Reagent with 4′,6-Diamidino-2-Phenylindole (DAPI) (Invitrogen, Sweden).

### Animal study

Male C57BL/6 mice weighed about 30 g were used in this study. They were injected intraperitoneally with 30 µg lipopolysaccharide (LPS) from Escherichia coli (Sigma-Aldrich, Sweden). Twenty-four hours later, the endothelial labeling of QDs was characterized in both *ex vivo* and *in vivo* experiments. For the *ex vivo* experiments, aorta from 5 LPS-treated mice and 4 control mice were dissected, washed with PBS, cut into small rings and fixed with 4% formaldehyde for 20 minutes. After fixation, aorta rings were incubated with VQDs or amino QDs in different concentrations (1.3 nM, 6.5 nM) for different time periods (1, 5 or 24 h). We found that 1.3 nM and 5 h incubation were optimal. After washing with PBS three times, aortic rings were opened longitudinally and mounted on glass slides using Prolong Gold Antifade Reagent with DAPI, with the endothelial layer facing the cover glass. For *in vivo* experiments, VQDs or amino QDs (10 pmol per animal) were injected via the tail vein: 2 control mice with VQDs, 4 LPS-treated mice with VQDs and 2 LPS-treated mice with amino QDs. Four hours later, mice were euthanized and aortas were collected. Aortic pieces were fixed, cut open and mounted, as described before. Blood was collected via cardiac puncture 10 minutes (2 mice) and 4 hours (6 mice) after QD injection. Blood smear on a microscopic slide was prepared. The animal experiments were approved by the local Animal Ethics Committee at the University of Göteborg (Ethical approval 116-2010).

### Experiment in tube

VQDs were mixed with the recombinant human VCAM-1 (R&D Systems, Sweden) in 10∶1 and 1∶1 molar ratios, respectively, and incubated for 5 hours at room temperature. VQDs were also incubated with 1% and 10% FBS, respectively, which were used as control samples. After incubation, a drop of the reaction mixture was added to a microscopic slide, dried then covered with a cover glass for spectra analysis using confocal microscope.

### Confocal microscopy

Leica TCS SP5 Confocal Microscope System was used to study the fluorescence spectra of QDs in different samples as well as the spatial distribution of QDs in the cells and tissues. All samples were excited with a 405 nm laser. Image signals were collected in the spectral range of 670–710 nm and analyzed using Leica Application Suite 2.02. The distributions of the QDs inside the cells were obtained using the 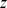-axis cross-sectional scan mode. Fluorescence spectra of QDs were acquired with a spectral resolution of 3 nm.

### Statistics

Data are presented as means
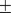
SD. Statistical comparisons were performed using ANOVA (SPSS 19.0) followed by Tukeys honestly significant difference test. A 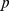 value less than 0.05 was considered statistically significant.

## Results and Discussion

### Conjugation of the QDs with VCAM-1 binding peptides

**Figure 2:**
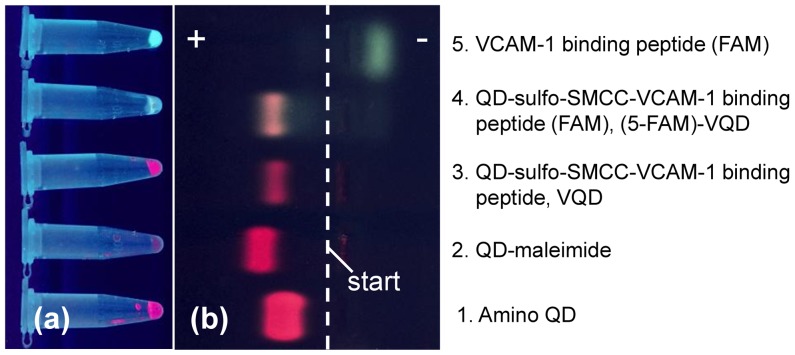
Gel electrophoresis results of different QDs. (a) QD samples in tubes after incubation and washing steps; (b) QD samples in gel after 1.5 h electrophoresis under UV light. The start line is shown as the white dash line and the positive electrode (+) is on the left side and negative electrode (−) on the right side.

The conjugation of QDs with VCAM-1 binding peptides was checked by electrophoresis experiments. Figure 2(a) shows different samples in tubes after incubation and washing steps, and Figure 2(b) shows different samples in the gel after 1.5 h electrophoresis under a voltage of 100 V. Here, the green fluorescence comes from 5-FAM and red from QDs. In Figure 2(b), the QD-maleimide sample (the 2nd sample) was loaded 4 fold extra as compared with other samples because the QD-maleimide solution was diluted with PBS during the filtration with NAP-10 column.

Amino QDs (the 1st sample) moved towards the positive electrode, indicating that these amino QDs are negatively charged. Similar phenomenon was reported before [31], [32]. One possible reason of this phenomenon is that the amino groups are deprotonated and have no charge within the pH range of the TAE buffer, while the surface of the amino QD is negatively charged because of the inner negatively charged polymer coating [32]. We also observed that QD-maleimide migrated faster than amino QDs towards the positive electrode.

The conjugation products, VQDs and (5-FAM)-VQDs (the 3rd and 4th sample, respectively), migrated slightly slower than QD-maleimide. The 5-FAM labeled VCAM-1 binding peptides (the 5th sample), on the other hand, migrated towards the negative electrode since all amino acids of the peptides were either neutral (V, P, Q, G, C) or positively charged (H, K, R) [32]. This also explains why VQDs move slightly slower than QD-maleimide. Note that the colour of the 4th sample is different from the 3rd sample since the green fluorescence from 5-FAM migrated together with the red fluorescence of QDs, indicating that 5-FAM labelled VCAM-1 binding peptides were successfully conjugated to amino QDs. Moreover, in the 4th sample, there was no band at the position of 5-FAM labelled VCAM-1 binding peptides so that un-conjugated 5-FAM labelled VCAM-1 binding peptides were successfully removed.

**Figure 3:**
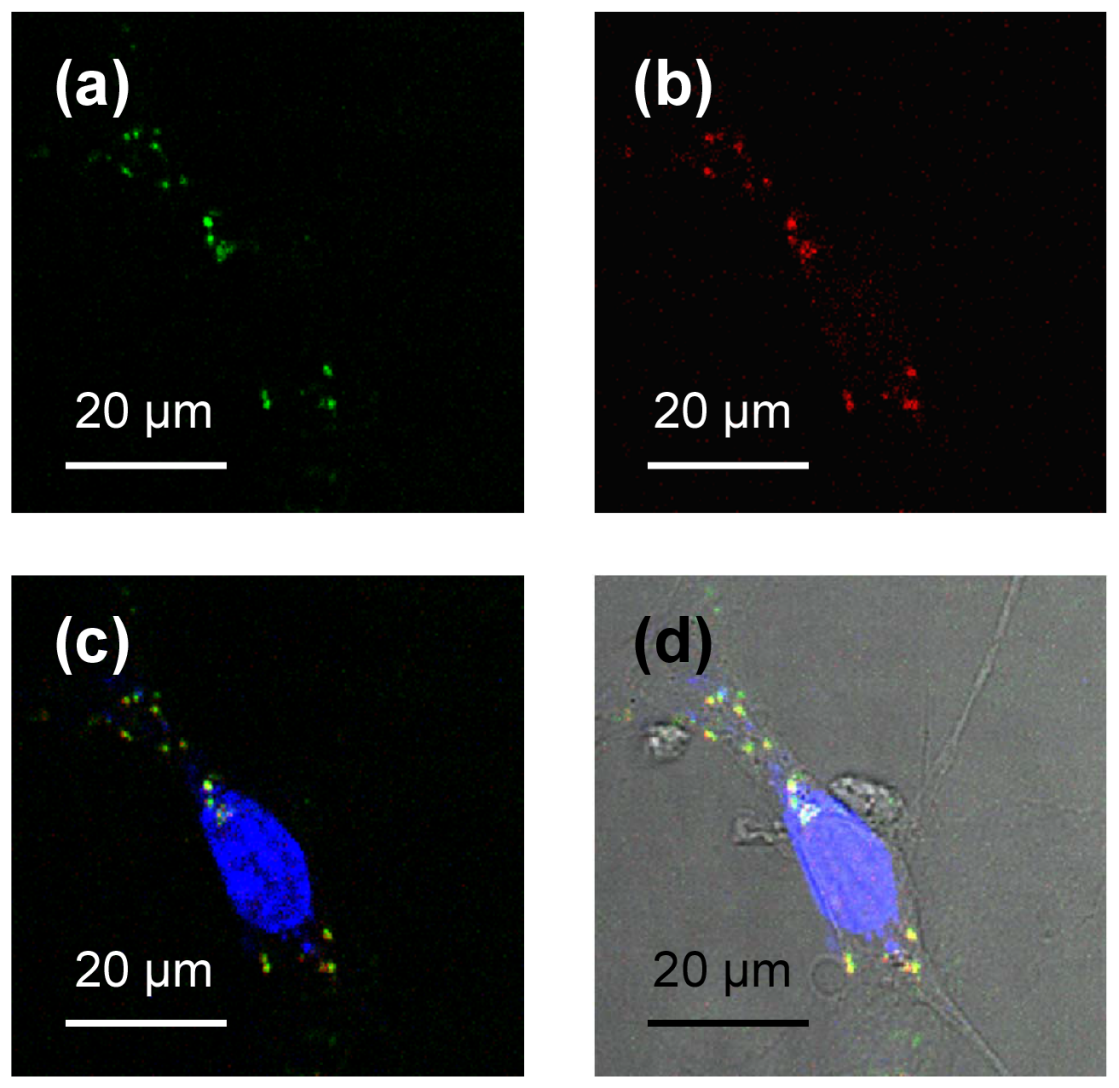
Confocal images of mouse endothelial cell after incubation with (5-FAM)-VQDs. (a) Green fluorescence from 5-FAM; (b) Red fluorescence from QDs; (c) Blue fluorescence from DAPI merged with (a) and (b); (d) A differential interference contrast image merged with (c).

It is therefore concluded that the conjugation of QDs with VCAM-1 binding peptides was successful. The successful conjugation between VCAM-1 binding peptides and QDs was further confirmed by *in vitro* cell study (Fig. 3). In mouse endothelial cells incubated with (5-FAM)-VQDs, the green fluorescence from 5-FAM co-localized with the red fluorescence from QDs.

### Increased QD uptake in VCAM-1-expressing endothelial cells labeled with VQDs

**Figure 4:**
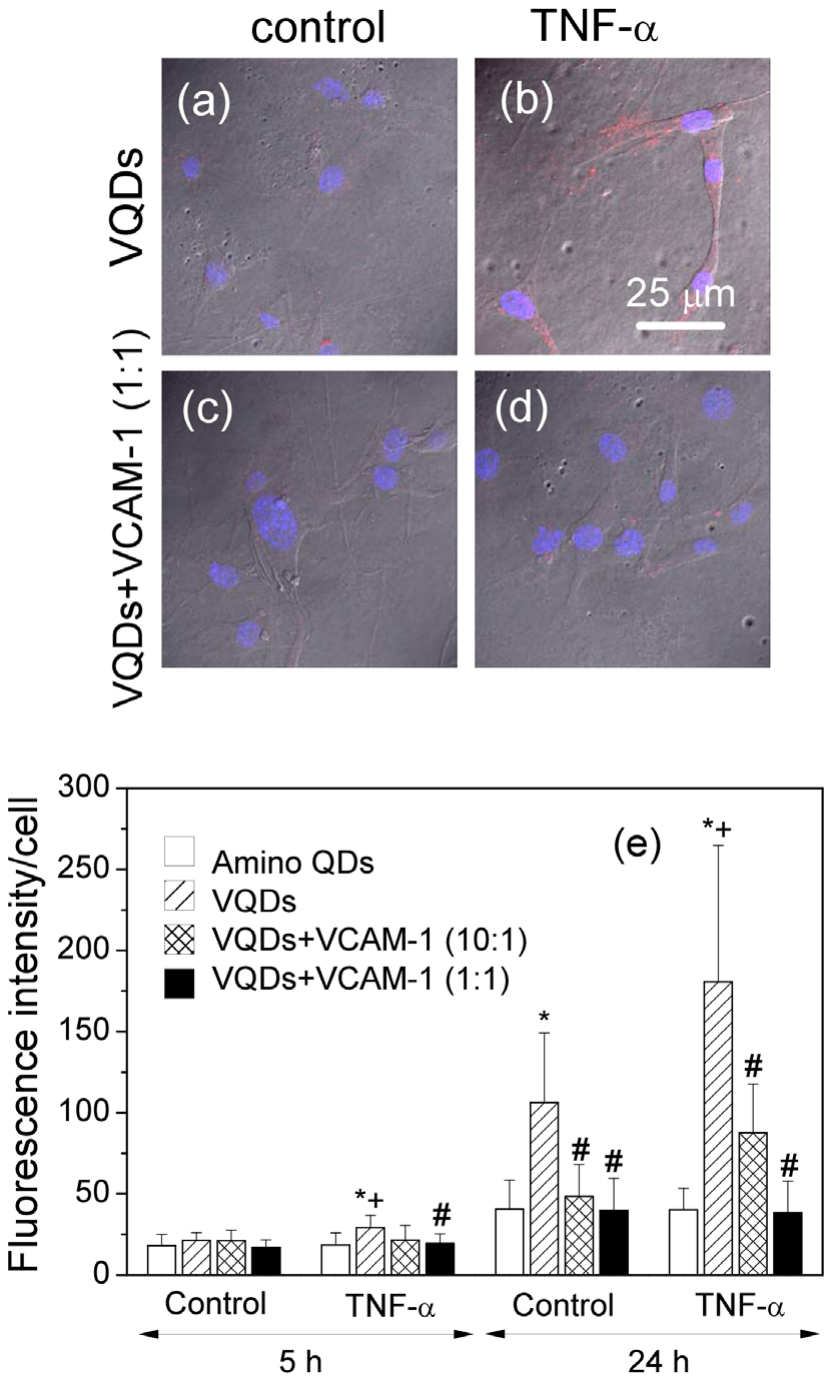
Conjugation with VCAM-1 binding peptide specifically increases the QD fluorescence signal in the VCAM-1 expressing endothelial cells. Confocal images of mouse endothelial cells: (a) Control cells incubated with VCAM-1 binding peptide functionalized QDs (VQDs) for 24 h; (b) TNF-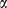 treated cells incubated with VQDs for 24 h; (c) Control cells incubated with pre-incubated mixture of VQDs and recombinant VCAM-1 (1∶1) for 24 h; (d) TNF-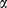 treated cells incubated with pre-incubated mixture of VQDs and recombinant VCAM-1 (1∶1) for 24 h. Blue signal comes from DAPI nuclei staining and red signal from QDs. (e) Quantification of fluorescence intensity. Values are means
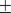
SD. 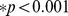 vs QD within the same treatment group and time point; 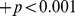 vs control VQD within the same time point; 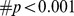 vs VQD within the same treatment and time point.

To study whether functionalization of QDs with VCAM-1 binding peptide could enhance uptake of QDs in VCAM-1-expressing cells, mouse endothelial cells were treated with TNF-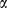 and then incubated with amino QDs or VQDs, respectively, for 5 and 24 hours. TNF-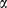 treatment increased VCAM-1 expression in these cells (data not shown). Figures 4(a) – (d) show confocal images of mouse endothelial cells after incubation with VQDs for 24 h. The QD signals were distributed in the cytoplasm, appearing as small bright spots. The QD fluorescence was stronger in the TNF-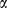-treated cells (Fig. 4(b)) than the control cells (Fig. 4(a)). Pre-incubation with recombinant VCAM-1 blocked the uptake of the VQDs in these cells (Fig. 4(c)-(d)), indicating the uptake was VCAM-1-specific. The quantification of the QD fluorescence intensity is shown in Figure 4. It was observed that the QD fluorescence intensity was higher in cells incubated with VQDs when compared to cells incubated with amino QDs. The QD fluorescence intensity was even higher when cells expressed more VCAM-1 upon TNF-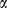 treatment than the control cells. The increment was already significant after 5 h incubation and became more pronounced after 24 h incubation. The incubation time needed to achieve relatively high level of the QD signal was longer than expected since it has been shown that internalization of VCAM-1 occurs rather rapidly [33]. Pre-incubation with recombinant VCAM-1 blocked cell uptake of VQDs so that the QD fluorescence intensity was decreased to a similar level as cells incubated with amino QDs. In a brief summary, our *in vitro* studies show that VQDs can be used to specifically image VCAM-1-expressing cells.

### Increased QD signals in the endothelium of mouse aorta labeled with VQDs

**Figure 5:**
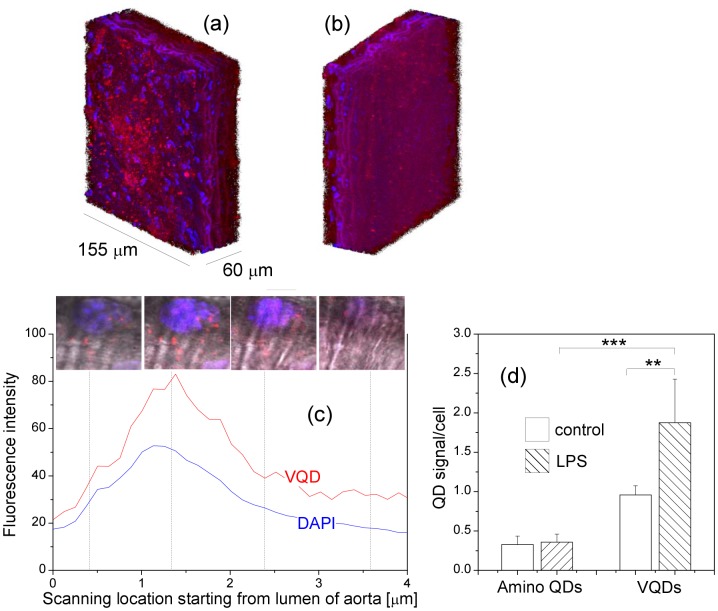
(a–b) Three-dimensional projection image of the aorta from one LPS-treated mouse. The aorta was labeled *ex vivo* with VQDs for 5 hours. The image was rotated so that the endothelial layer is shown in (a) and the adventitia is shown in (b). Blue signals come from DAPI nuclei staining and red from QDs. (c) Transversal distribution of fluorescence signals in mouse aorta. The scanning started from aortic lumen. The insets show confocal images in four different locations across the aorta. (d) Quantification of the QD signals in the endothelium of aorta from control and LPS-treated mice. Aortas were labeled *ex vivo* with amino QDs or VQDs for 5 hours, respectively. Values are means
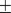
SD. 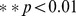, 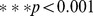.

To investigate whether VQDs can be used to detect VCAM-1-expressing endothelial cells in the aorta, we first performed *ex vivo* experiments where aortas from LPS-treated mice and control mice were prepared and incubated with amino QDs or VQDs, respectively. With the help of the scanning confocal microscope, we were able to study fluorescence signals in different layers in the aorta. We detected the first DAPI-labeled cell nucleus signal in the intimal layer, while only the fibrous structure was visible when scanning to the other side of the aorta (adventitia). Figure 5(a) – (b) show the three-dimensional projection images of the aorta from LPS-treated mouse after 5 h incubation with VQDs. Only the endothelial layer was labeled with VQDs(Fig. 5(a)). Deeper within the aorta the QD spots were rather sparse, and no signal was found in the adventitia (Fig. 5(b)). Quantification of fluorescence signals over the 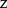-stack showed that bright QD spots appeared together with the first DAPI signals (Fig. 5(c)). Scanning towards to the middle of the aorta, the QD signal disappeared (data not shown). We observed a high and uneven auto-fluorescence in the aorta, which made it difficult to compare QD signals between different tissues by recording the fluorescence intensity in the microscopic field. The QD signal was quantified by counting bright QD spots that appeared as 1 µm spherical dots and cell nuclei in a randomly selected microscopic field. For each aorta, 4–6 microscopic fields were evaluated. Our data showed that in aortas incubated with amino QDs, the level of the QD signal was low and did not differ between the control and LPS-treated mice (Fig. 5). Compared with aortas incubated with amino QDs, incubation with VQDs resulted in a much strong QD signals: an increment of 192% (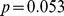) in the aorta of control mice and 422% (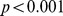) in the aorta from LPS-treated mice. Furthermore, when incubated with VQDs, aorta from LPS-treated mice had a significantly higher level of QD signals than the controls (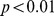).

**Figure 6:**
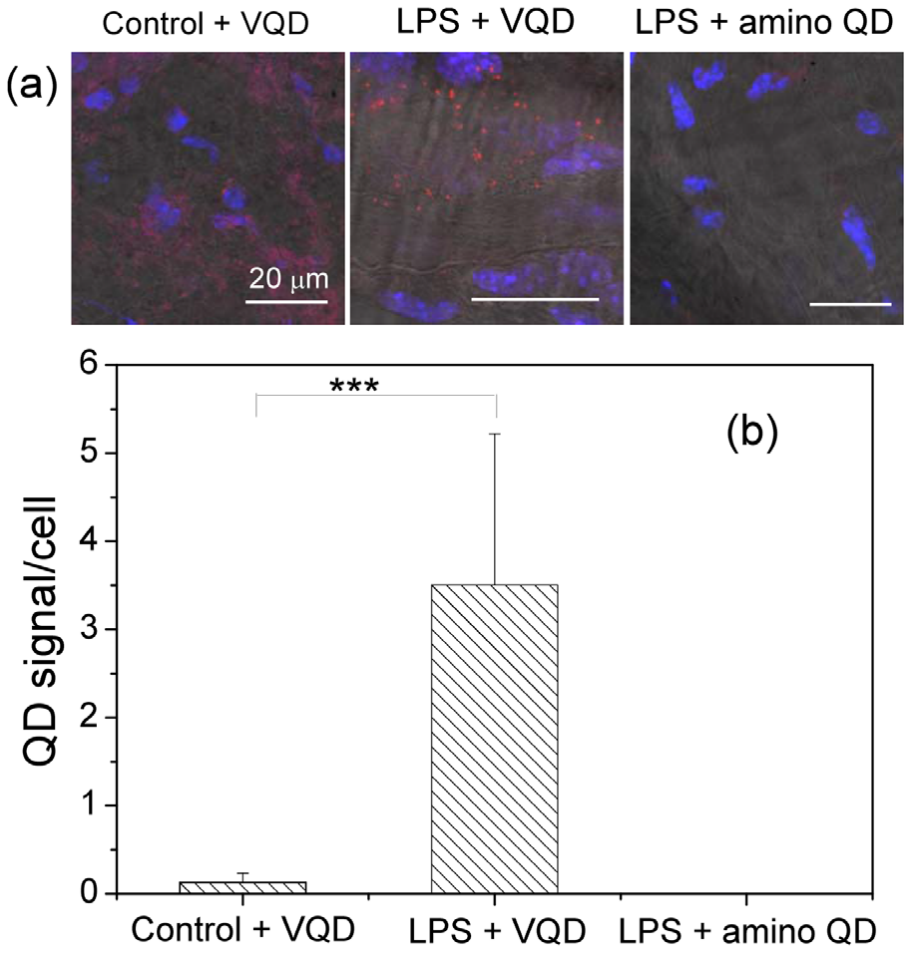
(a) Confocal images of the endothelial layer of mouse aortas labeled *in vivo* with 10 pmol VQDs or amino QDs, respectively. Red fluorescence from QDs; Blue fluorescence from DAPI. (b) Quantification of the QD signals in the endothelium of aorta from control and LPS-treated mice. Values are means
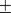
SD. 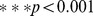.

Next we performed *in vivo* studies where VQDs or amino QDs were injected to animals via the tail vein and aortas were collected 4 hour later for evaluation. Our data showed that in mice received VQDs, QD signals in the aorta from the LPS-treated mice were much stronger than controls (Fig. 6). The QD signal was found in the endothelial layer of the aorta, but not in the media or adventitia, similar to what we observed in the *ex vivo* study (data not shown). No QD signal was detected in the aorta of LPS-treated mice that received amino QDs.

### Blue shift of fluorescence peak of VQDs upon binding to cell surface

**Figure 7:**
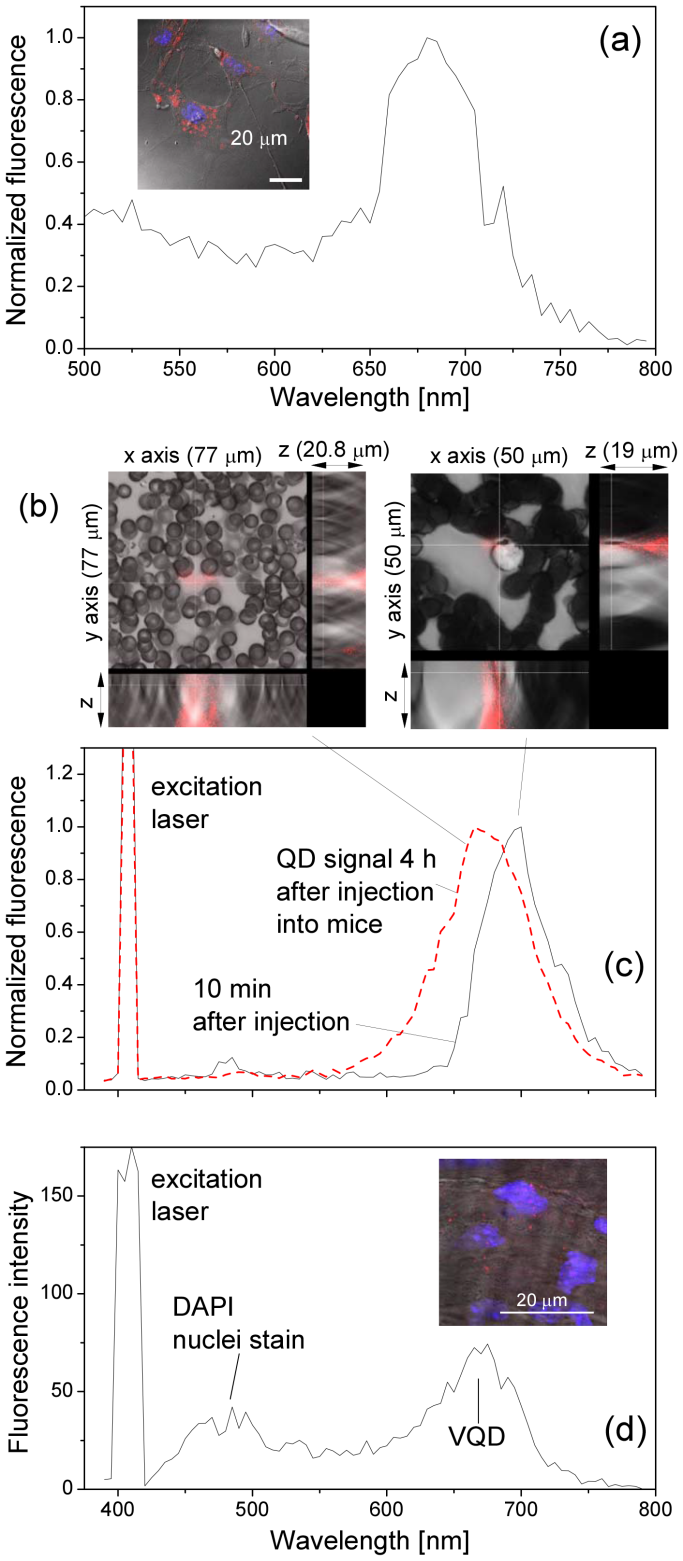
Blue-shift of the QD fluorescence peak from (a) cultured endothelial cells, (c) blood smear and (d) endothelial layer of mouse aorta. Inset in (a): Confocal image of TNF-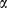 treated-endothelial cells incubated with VQDs for 24 h. (b): Three-dimensional confocal images of blood smears took at 10 minutes (left) and 4 hours (right) after VQD injection. The cross of two axes is the point where QD spectra in (c) were measured. Inset in (d): Confocal image of the aortic endothelial layer of LPS-treated mouse 4 hours after VQD injection. Blue signal is DAPI nuclei staining and red signal from QDs.

To ensure that the fluorescence signal came from QDs, fluorescence spectra of bright spots were always measured. We observed that the fluorescence peak of QD spots located inside the TNF-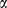 treated endothelial cell was significantly blue-shifted (Fig. 7(a)). On blood smear, QD spots were rare. We observed that at 4 hours after injection of VQDs, QD fluorescence was found in the close vicinity to blood cells, the QD fluorescence peak became broader, and most significantly, it was also blue-shifted from about 700 nm to about 670 nm when compared with QD fluorescence spectra from blood sample collected at 10 minutes, where QD fluorescence was distributed between blood cells (Fig. 7(b)). Note that the peak intensities of the two spectra were normalized in order to visualize clearly the blue-shift. Furthermore, we observed that in the aorta from LPS-treated mice that received VQDs, the QD fluorescence peak from the endothelial layer was at about 670 nm, i.e., the QD fluorescence peak was similarly blue-shifted about 30 nm (Fig. 7(c)).

### Blue shift of fluorescence peak of VQDs upon binding with VCAM-1 in tube

**Figure 8:**
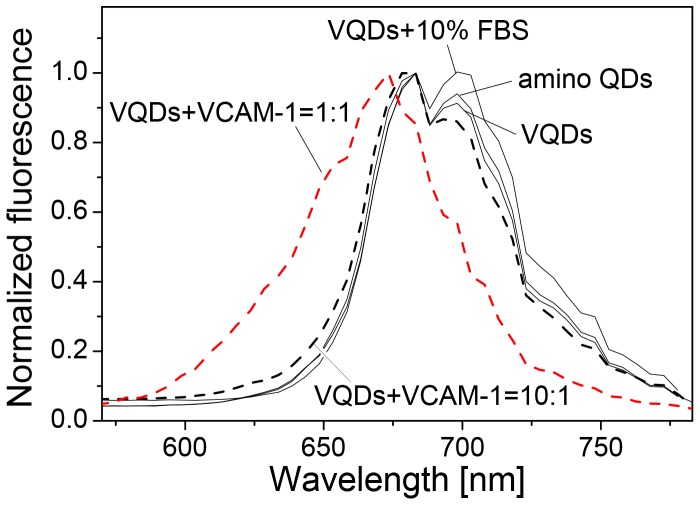
Blue-shift observed after incubation in tube. Fluorescence spectra of amino QDs, VQDs, VQDs + 10% FBS, VQDs + VCAM-1 (10∶1) (black dash line) and VQDs + VCAM-1 (1∶1) (red dash line) are shown.

As demonstrated earlier, the blue-shift of the QD fluorescence peak may be caused by different circumstances such as surrounding media or specific binding to other molecules such as DNA hybridization. To investigate whether the blue-shift observed here is caused by direct interactions between VQDs and VCAM-1 at cell surface, VQDs were incubated with the recombinant VCAM-1 in tube. A similar blue-shift in the QD fluorescence peak was observed after VQDs were incubated with the recombinant VCAM-1 in 1∶1 molar ratio, but not in 10∶1 ratio (Fig. 8). No clear blue-shift was observed when VQDs were incubated with FBS, where unspecific bindings with different molecules in serum may occur. Furthermore, no blue-shift was observed in the fluorescence spectrum of VQDs as compared with that of amino QDs (Fig. 8). Thus, the observed blue-shift is indeed related to the direct binding between VQDs and the VCAM-1. Given that VQDs were found inside cells, we cannot rule out the possibilities that internalization of VQDs may also contribute to the blue-shift phenomenon. Furthermore, we cannot rule out the possibility that other endothelial cell surface molecules may interact with VQDs in a similar way as VCAM-1, thus contributing to the blue-shift. Note that there seemed to exist two peaks in the fluorescence spectra of amino QDs, VQDs, and VQDs in 10% FBS. This was somehow intrinsic in amino QDs purchased from Invitrogen.

### Discussion of the blue shift

Figures 7 and 8 show that the QD fluorescence peak blue-shifts from about 696 nm (1.782 eV) to 670 (1.851 eV) nm after the direct binding between VQDs and the VCAM-1, i.e., an energy shift of 68 meV. Here the peak wavelengths of 696 and 670 nm were obtained by fitting the fluorescence peaks in Figs. 4, 5 and 6 by Lorentz peaks since the obtained QD fluorescence peaks are not smooth.

A similar but much small blue-shift (28 meV) of the fluorescence peak was observed in our early work when un-functionalized CdSe QDs (coated directly with 3-mercaptopropionic acid) were up-taken by EPCs [23]. Low pH, oxidizing molecules, and high enzyme concentration can degrade the coating of the QDs so that the QD size is reduced and the fluorescence peak blue-shifts [34]. However, the Invitrogen QD used in this work is coated by an amphiphilic polymer inner coating, providing a water-soluble surface, which is then covalently modified with a functionalized polyethylene glycol (PEG) outer coating. In other words, the surface coating is very robust. Furthermore, since the blue-shift we observed in this study also occurred *in vitro* by simply adding VCAM-1 molecules to VQDs in aqueous solution, coating degradation is very unlikely to be the principal cause of the observed blue shift in the current work.

The energy of the photon from QD fluorescence is the sum of (1) energy bandgap of the QD material, (2) quantum confinement energies of electrons in the conduction band (CB) and holes in the valence band (VB), minus the Coulomb interaction energy between the CB electron and VB hole. Our early theoretical studies show that the energy bandgap of the QD material does not depend significantly on the QD environment such as surface ligand modifications [35], [36] and the presences of ions [24]. On the other hand, we can speculate that physical and/or chemical interactions among the atoms at the surface of the QD and the QD environment, such as Cd atoms with lipids and proteins, S atoms with various ions inside the cell, as well as the ligand-receptor binding, alter the electronic structure at the QD surface so that the effective radius of the quantum confinement becomes reduced which increases the quantum confinement energies of the CB electrons and VB holes. It is easy to show that for the CdSe QD under investigation, a monolayer reduction at the surface of the QD results in a quantum confinement energy increase of about 44 meV (for detailed theoretical analysis, see Ref. [35]), which is only about a half of the experimentally observed blue shift of 68 meV.

Another possible contribution to the observed blue shift is the Coulomb interactions among VCAM-1, the CB electron, and the VB hole due to the binding of the VQDs with VCAM-1. As shown in Fig. 2, amino QDs are negatively charged, while VCAM-1 binding peptides are positively charged. Theoretical estimation shows that the CB electron and VB hole in the amino QD are not significantly perturbed by the VCAM-1 binding peptides due to the uniformly distributed negative charges at the polymer-coated QD surface. On the other hand, VCAM-1 are proteins which are negatively charged [37]. When VQDs were incubated with the recombinant VCAM-1 at a 1∶1 molar ratio, the CB electron and VB hole are under the influence of the Coulomb potential from the negative charge of the VCAM-1, which is normally referred to as the quantum Stark effect, see for example, Ref. [38]. The energy separation between the CB electron and VB hole states is reduced, while at the same time, the Coulomb interaction between the CB electron and VB hole is also reduced due to the spatial separation of the CB electron and the VB hole (the CB electron is pushed away from the VCAM-1, while the VB hole is attracted to the VCAM-1). Because of its large effective mass, the VB hole is much attracted by the negative charge at the QD surface so that the reduction in the Coulomb interaction between the CB electron and VB hole exceeds the reduction in the energy separation between the CB electron and VB hole states. We eventually obtain an increased fluorescence energy of the QD, similar to the observed blue shift in the fluorescence peak of the QD when the VQDs were incubated with the recombinant VCAM-1 at a 1∶1 molar ratio. By using the same energy band structure of Ref. [35], we obtained that the theoretical expectation of the blue shift is only about 15 meV.

On the other hand, the following extreme situation can also be speculated. The VB hole in the QD becomes tightly bound to the negative charge of the VCAM-1 when VQDs are bound to the VCAM-1, so that the Coulomb interaction between the CB electron and the VB hole is significantly diminished because of the spatial separation between them. For the QDs from Invitrogen, the diameter of the QD is about 15 nm so that the Coulomb interaction is about 80 meV [35]. This is very close to the experimentally observed blue shift. This can be very likely since the binding between the VQDs and VCAM-1 is very stable, as demonstrated by our *in vivo* and *in vitro* experiments. However, this scenario also means a much decreased QD fluorescence intensity since the CB electron and VB hole are now spatially separated so that their radiative recombination must be weak. This does not agree with the experimental observation.

Most probably the observed blue shift is a collective combination of the above effects.

## Conclusion

In this study, we developed a protocol for producing VCAM-1 binding peptide-functionalized QDs (VQDs). We demonstrated that the VQDs can be used to visualize TNF-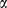-treated endothelial cells *in vitro* as well as inflamed endothelium *ex vivo* and *in vivo*. We found that the direct interaction between VQDs and VCAM-1 caused a significant blue-shift in the QD fluorescence peak, and this explains, at least partly, the blue-shift phenomenon observed *in vitro* and *in vivo*. The blue-shift phenomenon can be very useful for applying VQDs to specifically detect VCAM-1 *in vivo*, allowing imaging of early vascular changes.
